# Hierarchically Structured Biodegradable Microspheres Promote Therapeutic Angiogenesis

**DOI:** 10.1002/adhm.202401832

**Published:** 2024-09-11

**Authors:** Eseelle K. Hendow, Francesco Iacoviello, Mar Casajuana Ester, Caroline Pellet‐Many, Richard M. Day

**Affiliations:** ^1^ Centre for Precision Healthcare UCL Division of Medicine University College London Gower Street London WC1E 6BT UK; ^2^ Electrochemical Innovation Lab UCL Department of Chemical Engineering University College London Roberts Building London WC1E 7JE UK; ^3^ Department of Comparative Biomedical Sciences Royal Veterinary College 4 Royal College Street London NW1 0TU UK

**Keywords:** macrophages, microspheres, neovascularization, therapeutic angiogenesis, thermally induced phase separation

## Abstract

Promoting neovascularization is a prerequisite for many tissue engineering applications and the treatment of cardiovascular disease. Delivery of a pro‐angiogenic stimulus via acellular materials offers several benefits over biological therapies but has been hampered by interaction of the implanted material with the innate immune response. However, macrophages, a key component of the innate immune response, release a plurality of soluble factors that can be harnessed to stimulate neovascularization and restore blood flow to damaged tissue. This study investigates the ability of biodegradable poly(D,L‐lactic‐co‐glycolic acid) (PLGA) microspheres to restore tissue perfusion in a hind limb model of ischaemia. Microspheres exhibiting a hierarchical porous structure are associated with an increase in blood flow at day 21 post‐implantation compared with solid microspheres composed of the same polymer. This corresponds with an increase in blood vessel density in the surrounding tissue. In vitro simulation of the foreign body response observed demonstrates M2‐like macrophages incubated with the porous microspheres secreted increased amounts of vascular endothelial growth factor (VEGF) compared with M1‐like macrophages providing a potential mechanism for the increased neovascularization. The results from this study demonstrate implantable biodegradable porous microspheres provide a novel approach for increasing neovascularization that could be exploited for therapeutic applications.

## Graphical abstract

Promoting neovascularization is an unmet need for cardiovascular disease. Delivery of a pro‐angiogenic stimulus from implantation of biodegradable porous microspheres is shown to result in restoration of tissue perfusion in a hind limb model of ischaemia. M2‐like macrophages incubated in vitro with the porous microspheres secrete increased amounts of VEGF providing a potential mechanism for the increased neovascularization.

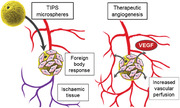

## 1 Introduction

An adequate vascular supply is essential for maintaining tissue viability. This is evident in a wide variety of physiological processes that occur naturally during growth, development, and wound healing through to the engineering of new tissue associated with emerging advanced therapies and regenerative medicine that are intended to repair or replace damaged or diseased tissues.^[^ ^1^, ^2^ ^]^ Many different biomedical approaches have been explored for initiating or improving tissue vascularisation, including delivery of pro‐angiogenic growth factors, cells, and more recently biomaterials.^[^ ^3^, ^4^, ^5^, ^6^, ^7^, ^8^ ^]^ Whilst limited success has been reported with these approaches using in vitro models, translation into clinically useful products has often been tempered by the impact of the host innate immune response, particularly the “foreign body response”, to the cellular and acellular components that often give rise to inflammation, halted wound healing, fibrotic encapsulation, and implant failure.^[^ ^9^ ^]^ As a result of this response, much attention has historically focussed on developing materials‐based approaches that minimize activation of the immune system in the hope that it improves biocompatibility of the implanted material and consequently increases the likelihood of a successful outcome.

Recent advances in understanding the immune system and the specific components that interact with implanted materials has led to a paradigm shift toward exploring whether aspects of the innate immune system that play key roles in the foreign body response can be harnessed and used for beneficial effect.^[^ ^10^, ^11^, ^12^, ^13^, ^14^ ^]^

Upon interaction with a foreign body, such as an implanted material, monocytes associated with the immune response are recruited to the implant and differentiate into macrophages, driving the inflammation further. Macrophages that interact with the surface of the material fuse to form foreign body giant cells.^[^ ^15^ ^]^ The extent of the ensuing tissue inflammation is dependent on the polarisation of macrophages, historically broadly categorized into type 1 macrophages (M1) or type 2 macrophages (M2), which is controlled by paracrine activity of the released cytokines and growth factors.^[^ ^11^, ^14^, ^16^ ^]^ M1 macrophages are associated with the secretion of pro‐inflammatory cytokines including interleukin (IL)‐1, −6 and‐23 that drive the inflammatory response and ultimately result in fibrous scar tissue formation or infection.^[^ ^17^ ^]^ M2 macrophages are associated with the secretion of pro‐angiogenic factors, including IL‐8, fibroblast growth factor (FGF), platelet‐derived growth factor (PDGF), and vascular endothelial growth factor,^[^ ^18^ ^]^ and can be further categorized into four subtypes (M2a, M2b, M2c, and M2d).^[^ ^18^, ^19^ ^]^ M2 macrophages are associated with resolution of the inflammatory response and onset of angiogenesis.^[^ ^20^ ^]^

Increasing evidence supports the concept that biomaterials could be used influence the foreign body response, including the manipulation of macrophage polarisation from a pro‐inflammatory type 1 macrophage (M1) phenotype toward a more reparative type 2 macrophage (M2) phenotype.^[^ ^11^, ^14^, ^16^ ^]^ Previous investigations have demonstrated the foreign body response can be influenced through the physical features of materials, including surface roughness,^[^ ^12^ ^]^ porosity,^[^ ^21^ ^]^ stiffness,^[^ ^22^ ^]^ hydrophobicity,^[^ ^23^ ^]^ and surface chemistry.^[^ ^14^ ^]^ Moreover, the porosity of a material appears to significantly modulate the response, with materials exhibiting larger pores being associated with increased levels of vascularisation compared with materials containing smaller pores, a response attributed to the presence of M2 macrophages secreting pro‐angiogenic factors.^[^ ^24^, ^25^ ^]^ Interconnectivity of the porous network may also improve nutrient and oxygen transfer, supporting tissue viability and the stabilization of newly formed vascular structures.^[^ ^26^ ^]^

The release of endogenous factors from the foreign body response associated with implanted materials is clearly capable of inducing tissue vascularisation but control of this response is essential to capture a beneficial effect and avoid progression toward chronic inflammation and the possible risk of fibrous encapsulation of the implant. One approach for achieving a controlled response would be to employ implantable bioresorbable materials that exhibit physical features capable of stimulating a limited foreign body response that resolves once the material is fully degraded.^[^ ^27^ ^]^ Microspheres provide an ideal format for minimally invasive delivery and can be fabricated from a variety of materials, including poly(alpha‐hydroxy esters), such poly(D,L‐lactic‐co‐glycolic acid), which have an excellent track record in clinical use and exist in a range of compositions that allows tailoring of the rate of degradation based on their ratio of lactide:glycolide. Furthermore, the polymer is amenable to a variety of microsphere fabrication processes, including thermally induced phase separation (TIPS) that provides a method for introducing a unique physical features that include a hierarchical and highly porous structure.^[^ ^28^ ^]^ It is hypothesized that PLGA microspheres capable of eliciting a controlled foreign body response associated with release of endogenous angiogenic factors could be used to treat clinical conditions, such as peripheral arterial disease (PAD), a condition that is becoming more prevalent with over 200 million people affected worldwide and for which there is currently no curative treatment.^[^ ^29^ ^]^ This approach could provide a new therapeutic strategy to promote angiogenesis capable of vascularizing ischemic tissue.

The aim of this study was to investigate the stimulatory effect on the foreign body response induced by the textured features associated with TIPS microspheres, paying particular attention to the pro‐angiogenic effect of the implanted material.

## 2 Results

### 2.1 Ultrastructural Features of TIPS Microspheres

**Figure 1:**
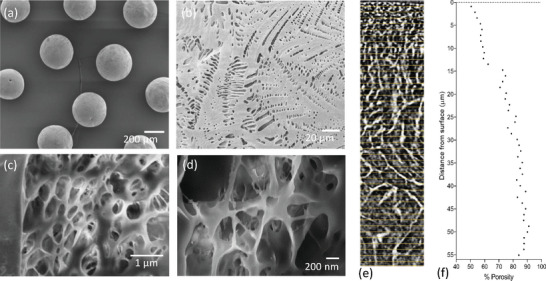
Scanning electron microscopy (SEM) of a) individual TIPS microspheres and b) higher magnification of the porous features on the surface of the TIPS microspheres at Day 0. c,d) Scanning electron microscopy of the internal structure of a TIPS microspheres at Day 0 following focused ion beam (FIB) milling showing the hierarchical internal structure. e) NanoCT imaging of a segment of a TIPS microsphere shows the denser surface region and increased porosity deeper inside the microsphere. f) Percentage porosity of a TIPS microsphere analyzed from NanoCT as a function of distance from the surface.

**Figure 2:**
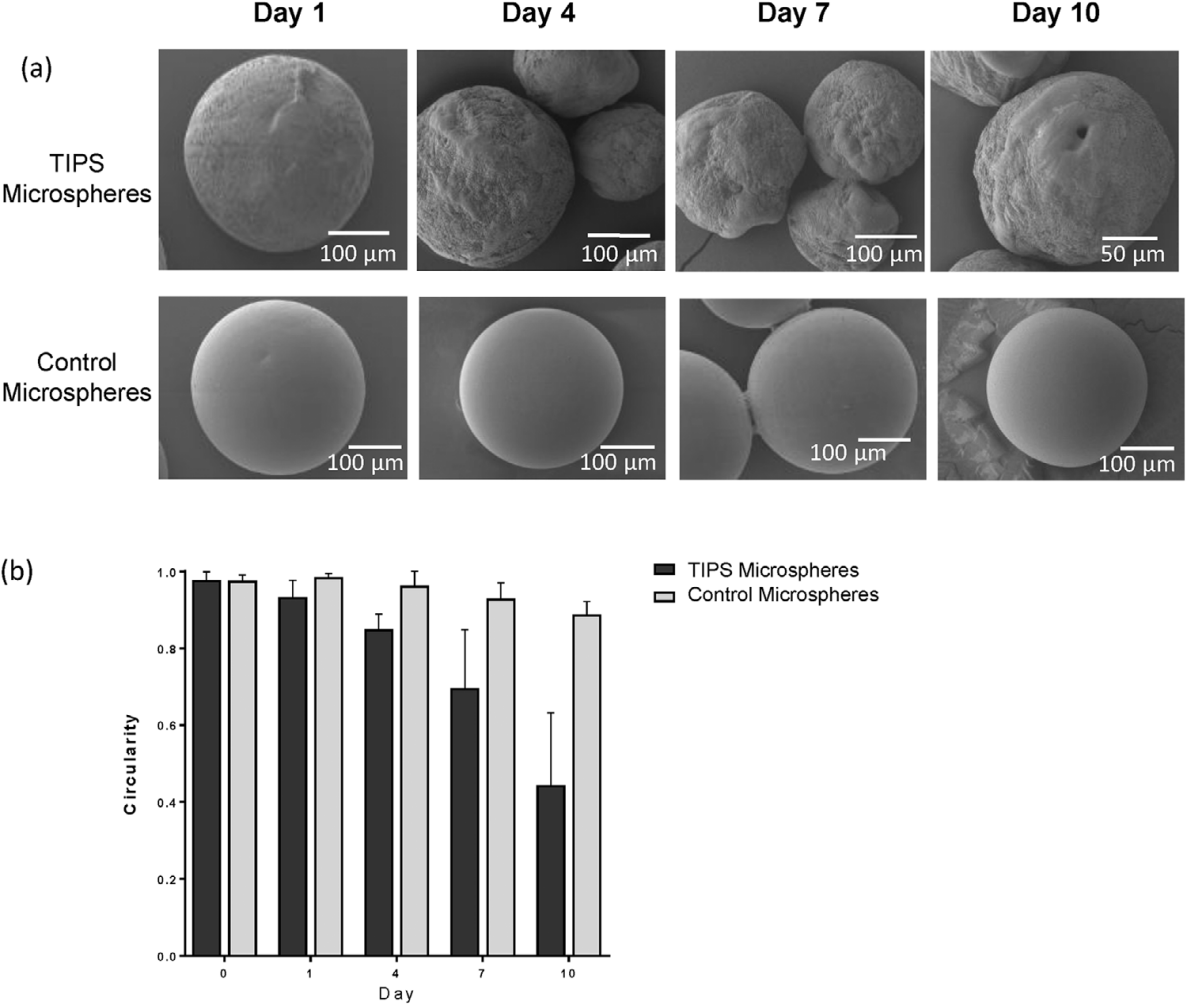
a) SEM images of TIPS and control solid microspheres at Days 1, 4, 7, and 10 simulated degradation show evidence of the TIPS microspheres degrading. All scale bars are 100 µm except for TIPS microspheres at Day 10 which is 50 µm. b) Circularity measurements of TIPS and control solid microspheres at Days 0, 1, 4, 7, and 10, calculated from SEM images using ImageJ software. Circularity was significantly reduced between TIPS and control solid microspheres at day 10 (*p* < 0.0001; Data analyzed with 2‐way ANOVA with Tukey multiple comparisons correction).

Scanning electron microscopy (SEM) was used for comparative assessment of the surface topography of TIPS microspheres (**Figure** **1**a). The surface of TIPS microspheres was highly porous, with clusters of anisotropic pores ranging from ∼0.5 – 20 µm, arranged within feather‐like patterns (Figure 1b). The skin of the TIPS microspheres comprised of a dense less porous structure that transitioned into a region of increased porosity. FIB‐SEM imaging revealed a hierarchical porous internal structure containing trabeculae‐like columns of polymer partitioning multiple channels (Figure 1c,d). Nano computed tomography (CT) analysis indicated that the TIPS microspheres had a surface porosity of ≈50% that increased to ≈90% at a depth of ≈50 µm below the surface of the microspheres (Figure 1e,f). Solid microspheres composed of the same polymer had smooth, non‐porous surfaces (**Figure** **2**a). The mean diameter of the TIPS microspheres was 367.1 ± 34.08 µm and 306.53 ± 32.33 µm for the solid microspheres.

### 2.2 Simulated Degradation of TIPS Microspheres

An in vitro degradation study was performed to simulate changes to the physical properties of the two types of microspheres following in vivo implantation (Figure 2). As the TIPS microspheres degraded via hydrolysis they became progressively less circular, with microsphere circularity measurements reducing from 0.98 ± 0.02 at Day 1 to 0.44 ± 0.18 at Day 10 and becoming visibly smaller. The change in circularity of the solid control microspheres in the simulated degradation was less pronounced, changing from 0.98 ± 0.01 at Day 0 to 0.89 ± 0.03 at Day 10.

### 2.3 Implantation of TIPS Microspheres into Ischaemic Tissue

**Figure 3:**
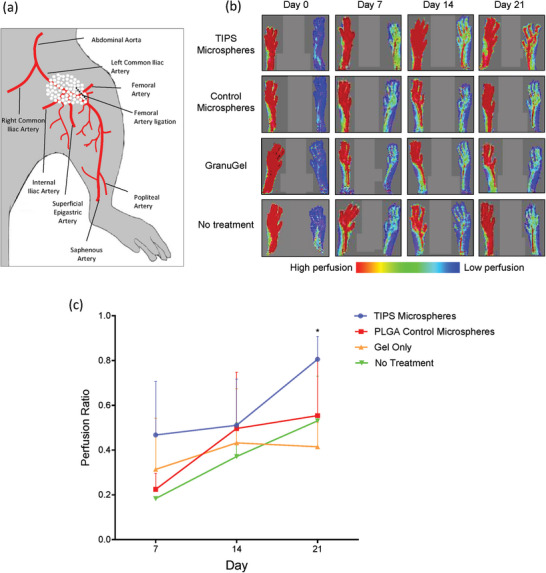
a) TIPS or control microspheres were delivered onto the tissue fascia in the region of vessel occlusion following unilateral femoral artery ligation in mice (black dotted line). The artery was ligated with a triple suture proximal to the deep femoral artery bifurcation. b) Laser Doppler imaging of the paws of mice that had undergone unilateral femoral artery ligation (paw on right) and implantation of TIPS microspheres, control solid microspheres, delivery vehicle (GranuGel) or no treatment. Images were acquired immediately after surgery (Day 0) and at Days 7, 14, and 21. c) Quantification of laser Doppler perfusion values in the paws of mice that had undergone unilateral femoral artery ligation. Data are presented as the perfusion ratio at Days 7, 14, and 21 relative to the perfusion in the contralateral control limb paws (a value of 1.0 is equivalent to perfusion in untreated control limb) and show treatment with TIPS microspheres resulted in an increased perfusion ratio at day 21 relative to the contralateral control limb (n = 3 per group; data analyzed with 2‐way Anova with Geisser‐Greenhouse correction (* *p* = 0.015).

Blood perfusion into the paw on the treated limb was immediately reduced following vessel ligation in all treated mice compared with perfusion of the paw in the contralateral untreated limb (**Figure** **3**). An equivalent quantity of TIPS microspheres or control microspheres was delivered onto the tissue fascia overlying the occluded blood vessel. No gross macroscopic adverse events were observed during the 21 day test period following treatment with either type of microsphere.

The colors shown by the laser Doppler imaging in Figure 3b are qualitative for illustrative purposes. While it is not possible to directly attribute a quantitative figure to the color in terms of flow, the moorVMS‐LDF instrument provided Doppler values of perfusion, which were used to calculate the perfusion ratio shown in Figure 3c. Perfusion was considered to be fully restored when the perfusion ratio equals 1.0. At days 7 and 14 post‐implantation there was no statistically significant difference in the perfusion ratio between the mice treated with TIPS microspheres, control microspheres, or the control groups (no treatment and treatment with vehicle only) (Figure 3c). At day 21 post implantation the group that received TIPS microspheres had a significantly increased perfusion ratio (0.8 ± 0.1 perfusion ratio) compared with all no treatment groups.

**Figure 4:**
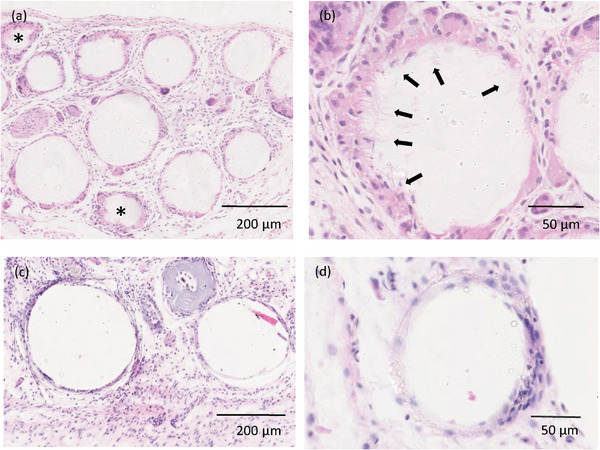
Haematoxylin and eosin staining of tissue explanted from the site of microsphere implantation in the hind limb ischaemia model at day 21. a) Tissue containing TIPS microspheres show a different pattern for the distribution of giant cells at the surface of the microspheres compared with solid microspheres composed of the same polymer (c). Asterix (*) indicates microspheres cut toward their cap. b) Higher magnification reveals cells from the foreign body reaction infiltrating the porous surface of TIPS microspheres (arrows), which was not visible with the solid microspheres (d).

**Figure 5:**
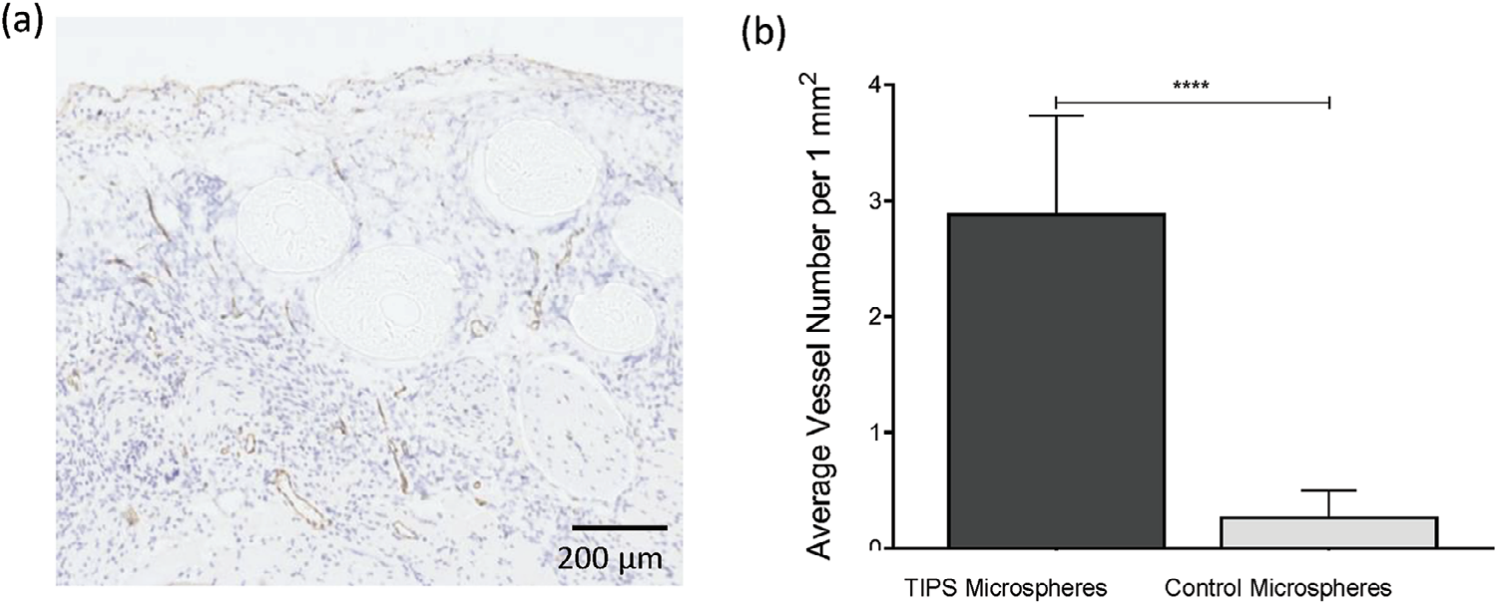
a) Von Willebrand Factor (VWF) staining of blood vessels in the tissue surrounding the implanted TIPS microspheres at day 21. b) Quantification of the number of vessels positively stained for VWF surrounding the implanted TIPS and control solid microspheres at day 21. The number of vessels was counted by three independent observers. (n = 3 mice per group, five fields of view per sample. Data analyzed using Mann–Whitney test **** *p* < 0.0001).

Histological analysis of tissue explanted from the implant site at day 21 revealed that the TIPS microspheres and solid microspheres remained at the implant site (**Figure** **4**). The TIPS microspheres showed signs of degradation and structural deformation, like that observed in the in vitro degradation study. The implanted solid microspheres showed less evidence of degradation and deformation of their circular structure, which also reflected the in vitro degradation study. Both groups of implanted materials were surrounded by soft granulation tissue consisting of loose connective tissue and blood vessels with an inflammatory cell infiltrate. Cell interaction with host tissue was closely apposed with the surface of both groups of implanted material along with the presence of multinucleated giant cells. However, unlike the solid microspheres (Figure 4d) cells from the multinucleated giants cells were visibly infiltrating the surface of the TIPS microspheres (Figure 4c). Newly formed blood vessels were present within the neotissue that had formed between the microspheres (**Figure** **5**). Quantification of the blood vessels in the tissue surrounding the implanted microspheres revealed that there was a significantly higher number of blood vessels in the tissues surrounding the implanted TIPS microspheres compared with the tissue surrounding the implanted control solid microspheres .

**Figure 6:**
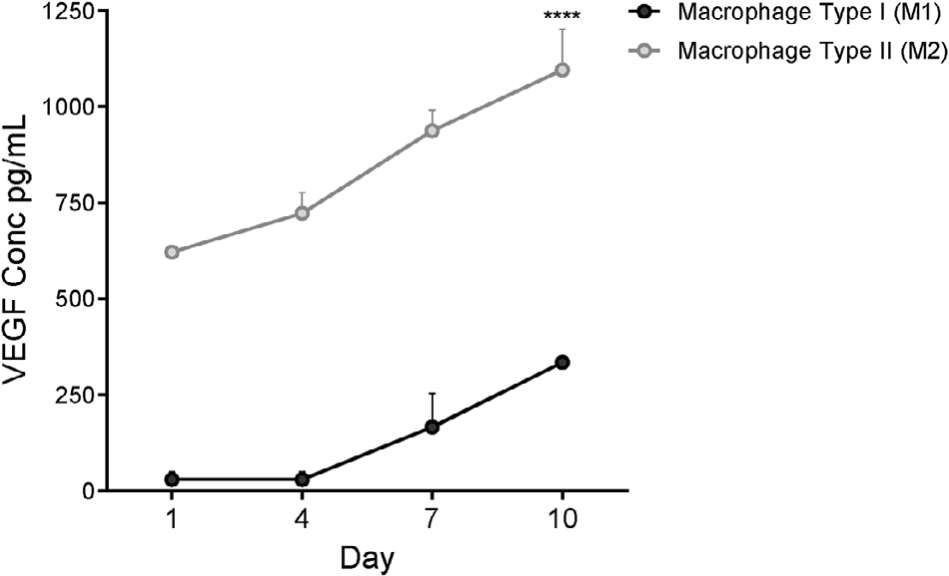
Vascular endothelial growth factor (VEGF) secretion from M1‐like and M2‐like macrophages incubated with TIPS microspheres at Days 1, 4, 7, and 10. VEGF measured in the supernatants was significantly higher from M2 macrophages. Data analyzed using 2‐way ANOVA with Sidak correction (**** *p* < 0.0001).

### 2.4 In Vitro Evaluation of Macrophage Interaction with TIPS Microspheres

THP‐1 cells were differentiated into an M0 macrophage‐like phenotype and subsequently further polarized into an M1 or M2 macrophage‐like phenotype using previously published protocols.^[^ ^32^, ^33^ ^]^ The increased level of IL‐12 p70 secretion in the supernatant, measured by ELISA, confirmed differentiation toward M1‐like macrophages (Figure S1, Supporting Information). To investigate the potential pro‐angiogenic effect of different macrophage phenotypes interacting with TIPS microspheres, cells polarized toward an M1‐ or M2‐like phenotype were incubated with TIPS microspheres. M2‐like cells secreted increased amounts of VEGF compared with M1‐like cells incubated with TIPS microspheres (**Figure** **6**).

## 3 Discussion

Highly porous microspheres were produced using a thermally induced phase separation fabrication method. A polymer concentration of 10% (w/v) was chosen to produce the microspheres since they are already undergoing clinical investigation for wound repair (ClinicalTrial.gov NCT03707769), which could facilitate easier translation into clinic, and have been previously shown to stimulate an increased angiogenic secretome from mesenchymal stromal cells in vitro compared with TIPS microspheres produced from 1% or 5% (w/v) PLGA.^[^ ^36^ ^]^ Imaging of the ultrastructure revealed a highly porous surface topography with pores arranged in a relatively dense surface skin that transitioned into a more open, hierarchically ordered, and highly porous internal structure, with pore sizes ranging from micro‐ to nano‐scale. The hierarchical structure of the TIPS material is potentially advantageous by simultaneously allowing cell infiltration while providing mechanical properties that resist compression. This type of structure mimics natural materials, such as bamboo fibers and bone, which possess hierarchical porous architectures along with superior mechanical properties. When implanted into a hind limb ischaemia model, both types of microsphere were well tolerated over the duration of the study, which is to be expected given the well‐established biocompatibility of PLGA and its use in a variety of clinically approved products, including from resorbable sutures and various drug‐device combinations for sustained delivery of active ingredients.^[^ ^37^, ^38^ ^]^ Histological analysis of the explanted tissue revealed both types of microspheres induced a moderate sterile inflammatory response that exhibited features typical of a foreign body reaction to biodegradable polymer microspheres.^[^ ^27^ ^]^ The initiation of the acute sterile inflammatory response and macrophage aggregation at the surface of both types of material was evident at 3 weeks post‐implantation, including fusion into foreign body giant cells indicative of “frustrated” phagocytosis. However, distinct differences were observed between the localized response to the two types of implanted materials. First, the band of inflammatory cells surrounding the TIPS microspheres with consistently thicker and more continuous compared with solid control microspheres composed of the same polymer. Quantification of the macrophages or their polarization in response to the implanted materials in vivo would be interesting; however, as can be seen from the histological images, the distribution of cells and capsule thickness appear to vary greatly between each implanted microsphere. This relates the plane of the tissue section relative to the geometry of the microsphere. For example, if the tissue is sectioned through the center of the microsphere, near its equator, the band of surrounding cells appears thinnest, whereas microspheres that have been cut toward their cap, the band of surrounding cells appear to be thicker. Second, the porosity of the TIPS microspheres appears to have allowed infiltration of the inflammatory cells into the peripheral structure of the microspheres. The solid structure of the microspheres in the control group prevented this from happening. The different interactions between the inflammatory infiltrate and the microenvironment presented by the porous structure of the TIPS microspheres might have contributed to differences observed. The porous structure may offer a provisional matrix providing physical cues for macrophages to infiltrate and undergo a phenotype transformation from a proinflammatory state to an anti‐inflammatory type called M2, which play a crucial role in promoting angiogenesis.^[^ ^39^ ^]^

Histological analysis was conducted to determine whether TIPS microspheres resulted in a difference in the level of vascularization within the local microenvironment of implanted microspheres versus the control microspheres composed of the same polymer. Changes observed in the inflammatory response between the two types of microspheres coincided with increased blood vessel density in the newly formed tissue surrounding implanted TIPS microspheres compared with the equivalent tissue regions in the groups receiving implantation of solid control microspheres. The vessels observed around the TIPS microspheres ranged in size and many included erythrocytes within the lumen indicating the presence of a functional vasculature. These observations correspond with the in vivo assessment of blood flow using laser Doppler imaging acquired before the tissues were explanted, which confirmed implantation of TIPS microspheres resulted in increased functional vasculature and increased blood perfusion of the ischaemic tissue. Significantly higher blood flow in the ischaemic limbs was measured at day 21 post‐implantation in the group treated with TIPS microspheres compared with the groups receiving solid microspheres, vehicle alone, or no treatment following induction of hind limb ischaemia, with the perfusion ratio approaching 1.0 that is equivalent to perfusion values in the untreated contralateral control limb. These data suggest the pro‐angiogenic effect in response to TIPS microspheres exceeds the endogenous angiogenic response that follows the ischaemic insult or the pro‐angiogenic activity of control microspheres or delivery vehicle. Further studies evaluating the perfusion values at time‐points beyond 21 days would clarify how long it takes for complete restoration of blood flow to occur in the treatment group.

Since both types of microspheres were composed of identical polymer, we postulate that the most likely cause for the differences in tissue vascularization and perfusion observed was due to the different physical structure of the microspheres. Post‐implantation, the TIPS microspheres exhibited features of biodegradation related to their porosity that included a loss of sphericity like that observed in the in vitro degradation study. Infiltration of host cells from the foreign body reaction into the surface layer of the implanted TIPS microspheres was visible indicating surface porosity remained during in vivo degradation. Cell infiltration by host tissue is known to aid physical integration of the microspheres with host tissue at the implant site, preventing off‐target migration that has been observed with implantation of non‐porous microspheres.^[^ ^40^ ^]^ This is particularly important when targeted therapy is required in conditions, such as peripheral arterial disease simulated in the current study, but the findings from the current study suggest porosity may also account for the pro‐angiogenic effect observed.

Unlike permanent, non‐degradable implants, TIPS microspheres undergo physicochemical changes as they degrade via hydrolysis, thus providing a temporary biophysical stimulus to promote neovascularization. Degradation‐induced changes to the microspheres are likely to include surface chemistry, hydrophilicity, and protein adsorption that play influential roles during the interaction between implanted materials and cells of the immune response.^[^ ^41^ ^]^ Since the current study was conducted up to 3 weeks the implanted biodegradable microspheres were still present at the end of study. Based on existing non‐clinical studies with this composition of TIPS microspheres, the microspheres would be expected to completely degrade over a period of several months before complete resorption of the implanted material and resolution of the foreign body reaction. The degradation rate of the microspheres is influenced by their porosity, size, and polymer composition (ratio of lactide:glycolide). Therefore, further studies are required to gain insight into how the increased tissue vascularisation and tissue infiltration observed in the current study influences the rate of microsphere degradation and eventual resolution of the foreign body reaction.

A dynamic interaction between the physicochemical changes to the implanted microspheres as it degrades and the interacting macrophages is likely to influence the immune response observed and this may contribute to increased vascularization and tissue perfusion observed with the porous TIPS microspheres. Macrophages exhibit plasticity in their phenotype depending on the surrounding microenvironment that can lead to a shift in cell polarisation from the pro‐inflammatory “M1” phenotype to the anti‐inflammatory/tissue “M2” macrophage phenotype reported to promote angiogenesis.^[^ ^39^, ^42^ ^]^ Cell infiltration into the surface of TIPS microspheres was clearly evident at 3 weeks post‐implantation. The ultrastructural porous features of TIPS microspheres observed using SEM and nanoCT provide unique conditions for cell infiltration and confinement within the material. The ability of macrophages, associated with the sterile inflammatory response, to interact with these distinct features might account for the observed greater pro‐angiogenic response to the TIPS microspheres in vivo compared with the same non‐porous material.

Increased vascularisation has been reported elsewhere to coincide with macrophage infiltration into implanted porous materials and a shift in macrophage polarisation. For example, cardiac implantation of porous sphere‐templated, poly(2‐hydroxyethyl methacrylate‐co‐methacrylic acid) (pHEMA‐co‐MAA) hydrogel scaffolds was associated with angiogenesis and increased neovascularisation occurring with implants exhibiting pores >20 µm, which coincided with a mixed population of macrophage phenotypes.^[^ ^26^ ^]^ Interestingly, a subsequent study by the same group indicated macrophages that had infiltrated implant pores were characterized as having a predominantly M1 phenotype compared with non‐infiltrating macrophage external to the implanted material.^[^ ^43^ ^]^ Although the specific role of macrophage sub‐types that infiltrated or were external to the implanted material and their secretion of pro‐angiogenic factors is not clear from these studies it is likely that a coordinated interaction of macrophages with mixed phenotypes is likely to deliver a secretome composed of key factors involved in angiogenesis, chemoattractants for stabilizing pericytes, anastomosis of sprouting endothelial cells and vascular remodeling.^[^ ^44^ ^]^

To explore how macrophage interaction with the porous microspheres might create a pro‐angiogenic microenvironment in vivo, we investigated the secretion of VEGF from THP‐1 macrophage‐like cells that were polarised in vitro toward an M1‐ or M2‐like phenotype when cultured on TIPS microspheres. M1‐ or M2‐like macrophages were introduced to a hanging drop plate. This approach was used to force the cells to interact with the microspheres being tested since there is no other surface for the cells to interact with. This was intended to reflect the interaction of macrophages with the microspheres observed in vivo. M2‐like macrophages secreted an increased quantity of VEGF compared with M1‐like macrophages, indicating that presence of TIPS microspheres might enhance the secretion of this key pro‐angiogenic growth factor. M2 macrophages are classified according to the secretory profile that includes anti‐inflammatory and pro‐angiogenic factors and are thus likely candidates for contributing to the increased tissue vascularisation and perfusion observed in response to TIPS microspheres following induction of hindlimb ischaemia in the current study.^[^ ^10^ ^]^ However, given the dynamic host/implant environment that exists as the microspheres degrade, the “M1” and “M2” classification used in conjunction with the in vitro modeling in the current study oversimplifies the likely balance between pro‐ and anti‐inflammatory / regulatory macrophages associated with the foreign body response and its contribution to the increased angiogenesis observed in response to implantation of the TIPS microspheres.

A cell‐based approach that involves acute sterile inflammation in response to apoptotic donor cells during cell transplantation therapy in immunologically active tissue (the “dying stem cell hypothesis”) has been proposed to modulate the local immune response and lead to improved clinical outcomes.^[^ ^45^ ^]^ The potential benefits of this phenomenon has recently been shown to contribute to improved heart function following cell therapy in mice after ischaemia reperfusion injury.^[^ ^43^ ^]^ The findings from this study suggest implantable, degradable biomaterials capable of eliciting a self‐limiting, sterile inflammatory response may provide an alternative acellular approach for achieving temporary selective stimulation of pro‐angiogenic macrophage subtypes. PLGA TIPS microspheres have not previously been investigated as a device to stimulate vascularization. PLGA is an advantageous polymer to investigate due to the relative ease for translating into clinic and when in a microsphere format enable minimally invasive delivery. We are not aware of any previous study looking at porous PLGA microspheres to stimulate vascularization. However, understanding the role played by macrophages in the pro‐angiogenic response observed requires a more “macrophage‐centered approach” that will characterize the composition of macrophages, as advocated elsewhere.^[^ ^10^, ^16^, ^46^, ^47^ ^]^

## 4 Conclusion

In conclusion, this proof‐of‐concept study supports the notion that temporary placement of biodegradable material provides a biophysical stimulus of the host immune system using that could be harnessed for innovative approaches in regenerative medicine. Further studies will be required to optimize the effect observed and obtain a better understanding of how macrophages are contributing to vascularization, and whether this can be utilized as a therapeutic approach to address clinical needs. The data presented may open up new areas of acellular materials research that can be further optimized to advance healthcare.

## 5 Experimental Section

### Fabrication of Microspheres

TIPS microspheres composed of 75:25 poly(D,L‐lactide‐co‐glycolide) (PLGA) were prepared as previously described.^[^ ^28^, ^30^ ^]^ PLGA PURASORB 7507 (75:25; inherent viscosity 0.70 dl/g) (Corbion, Amsterdam, Netherlands) was dissolved in dimethyl carbonate (Sigma Aldrich, Dorset, UK) overnight using magnetic stirring to produce a 10% (w/v) polymer solution. The polymer solution then was fed into a Nisco Encapsulator Unit (Nisco Engineering, Zurich, Switzerland; Frequency: 2.75 kHz, Amplitude: 70%) by a syringe pump (Pump 11, Harvard Apparatus, Kent, UK), at a constant flow rate of 2 ml min^−1^. The polymer droplets were formed using a 150 µm sapphire nozzle and collected in liquid nitrogen. Residual solvent was removed from the frozen polymer droplets by lyophilization for 48 hr. The dried PLGA TIPS microspheres were sieved to a size range of 250–355 µm and stored at room temperature in rubber stoppered glass vials under vacuum.

Solid microspheres composed of PLGA PURASORB 7507 at a size range of 250–355 µm were acquired from Phosphorex (PS300K, Phosphorex, USA).

### Hydration of PLGA Microspheres Pre‐Cell Attachment and In Vitro Degradation Study

TIPS microspheres and solid control microspheres were hydrated by placing 10 mg of microspheres into a 7 ml MesenPro RS reduced serum medium (12746‐012, ThermoFisher UK) supplemented 7% (v/v) absolute ethanol. The samples were incubated at 37 °C with constant rotation at 11 rpm for 18 hours.

An in vitro degradation study was conducted by aliquoting 30 mg of PLGA microspheres into glass vials. Phosphate‐buffered saline (pH 7.4) was added to each vial containing the microspheres to produce a final ratio of volume of the buffer solution of 10 mL:30 mg of microspheres. The vials were placed onto a tube roller and placed inside a dry incubator at 37 °C at 60 rpm constant rotation. At pre‐determined time‐points the vials containing the microspheres were rinsed twice with 10 mL analytical grade water. The samples were dried in a desiccator containing silica gel beads at room temperature to remove residual water before being analyzed by scanning electron microscopy.

### Ultrastructural Characterisation of Microspheres

Scanning election microscopy (SEM) was used to examine the ultrastructural surface features of the polymer microspheres. The samples were mounted onto aluminium stubs using carbon sticky tabs and sputter coated with gold/palladium alloy in an argon atmosphere. Samples were imaged using a Jeol 7401 high resolution field emission SEM. The size and circularity of the microspheres were analyzed from SEM images using ImageJ, with a circularity value of 1.0 indicating a perfect circle. Focused ion beam scanning electron microscopy (FIB) (Carl Zeiss XB1540 Cross Beam microscope) was used to visualize the internal structure of the TIPS microspheres by milling a 3 µm x 3 µm x 3 µm area into the surface of the microspheres followed by imaging using SEM.

Nano computed tomography (NanoCT) (Zeiss Xradia Ultra 810 nanoCT) was used to analyze the internal porosity of TIPS microspheres. A total of 721 projections were collected per 180° sample rotation with an exposure time of 10 seconds to provide a set of raw image data with an isotropic voxel resolution of 126 nm (camera binning of 2) and a field of view of 65 µm. In order to correct for the weak X‐ray absorption caused by the low density of the sample, Zernike phase contrast was used. The raw transmission images were reconstructed using an image reconstruction software package (Zeiss XMReconstructor, Carl Zeiss X‐ray Microscopy Inc., Pleasanton, CA), which employed a filtered back‐projection algorithm. To calculate porosity, the 3D reconstructed volume of the microsphere was segmented and analysed using Avizo Fire 9.2 software (Thermo Fisher Scientific, USA). A sub‐volume of 100 × 100 × 447 voxels (12.6 × 12.6 × 55.1 µm) was extracted and grayscale images were subsequently segmented into a binary dataset assigning different pixels to the pore phase and the solid polymer phase following a thresholding procedure. The process was performed on 44 slices at 1.13 µm intervals.

### In Vivo Assessment of Microspheres in Ischaemic Tissue

Microspheres were hydrated, washed with distilled H2O, and mixed with GranuGel (Convatec, UK) at a concentration of 200 mg ml^−1^. Hind limb ischaemia was induced in 2–3 month old female c57bl/6 mice by unilateral artery ligation.^[^ ^31^ ^]^ Under general anesthesia (1–3% isoflurane and 2 L min^−1^ O2), an incision along the center of left thigh was created exposing the common femoral artery. The artery was separated from the femoral vein and nerve and ligated with a triple suture proximal to the deep femoral artery bifurcation. 100 µL of microspheres in GranuGel was delivered around the occluded vessel bundle. Bupivacaine analgesia was administered intra‐muscularly and the wound was closed with 3–4 sutures. Immediately after surgery vessel occlusion was confirmed with laser Doppler imaging (moorVMS‐LDF, Moor Instruments, UK). The surgical procedure was repeated with the implantation of solid control microspheres in GranuGel (200 mg ml^−1^), vehicle (GranuGel) only, and no treatment following induction of hind limb ischaemia. Laser Doppler imaging was conducted on days 7, 14, and 21. At day 21 the mice were euthanized by overdose of CO_2_ followed by cervical dislocation and the leg muscles from the test site and contralateral control limb were harvested for analysis. All experiments were performed under a UK Home Office license (PLN: 70/7700), in compliance with the 1986 United Kingdom Home Office Animals (Scientific Procedures) Act and with the approval of University College London Local Ethics Committee.

### Histological Analysis

Explanted tissue was fixed in 4% neutral buffered formalin solution for 72 hours and processed into Paraplast X‐TRA low‐temperature paraffin wax (Sigma Aldrich). Tissue sections were stained with haematoxylin and eosin or immunostained for blood vessels using anti‐Von Willebrand Factor antibody at a dilution of 1:1000 (Ab11713, Abcam, UK) prior to imaging using a Nanozoomer 2.0‐HT (Hamamatsu, UK) digital slide scanner. To quantify the number of positively stained blood vessels in the tissue surrounding the site of implantation, three observers blinded to treatment conditions manually counted the quantity of positively stained blood vessels in 1mm^2^ regions of tissue in five fields of view per tissue section.

### In Vitro Interaction of Macrophage‐Like Cells with Microspheres

Human monocytic THP‐1 cells (kindly provided by Gilroy Lab, UCL) were seeded at a density of 4 × 105 cells/ml in RPMI‐1640 media (Sigma Aldrich) with HEPES modification and supplemented with 0.05 mm 2‐mercaptoethanol (Sigma Aldrich), 1 mm pyruvate (Sigma Aldrich), 200 mm L‐glutamine (Sigma Aldrich) and 10% heat inactivated foetal bovine serum (Life Technologies, UK). Cells were maintained in T75 flasks (ThermoFisher, UK) in suspension at a density of 1 × 106 cells/ml at 37 °C and 5% CO2. THP‐1 cells were differentiated into M0 macrophage‐like cells followed by polarisation into M1‐ or M2‐like macrophages following established protocols.^[^ ^32^, ^33^ ^]^ Polarisation into M1‐ or M2‐like macrophages after 48 h in differentiation medium was assessed by measuring human interleukin‐12 (IL‐12 p70) in the supernatants using ELISA (R&D Systems, UK). An M1 macrophage‐like phenotype was determined by the increased secretion of the M1 marker IL‐12 p70, whereas THP‐1, M0, and M2 cells do not express IL‐12 p70.^[^ ^34^, ^35^ ^]^

To investigate the interaction of macrophage‐like cells with TIPS microspheres, the cells and a single microsphere in 45 µL RPMI‐1640 media were loaded into individual wells of a hanging drop plate (3D Biomatrix, USA) at a seeding density of 1 × 104 cells/ml. VEGF secretion in the supernatants was measured using ELISA (R&D Systems, UK) at days 1, 4, 7, and 10.

### Statistical Analysis

The data were tested for normality using a Kolmogorov‐Smirnov test. If normally distributed, a parametric unpaired Welch Student t‐test was performed. If not normally distributed, a non‐parametric Wilcoxon Student *t*‐test was performed. For data with multiple independent variables 2‐way ANOVA with multiple comparisons was used. P values of <0.05 indicated statistical significance and were shown as *, with *P* < 0.01 = ** *P* < 0.001 = *** and *P* < 0.0001 = ****.

## Conflict of Interest

RMD is a Director of a company that produces 3D biomaterials for therapeutic applications.

## Supporting information



Supporting Information

## Data Availability

The data that support the findings of this study are available from the corresponding author upon reasonable request.
