# Supplementary material for: Hierarchically Structured Biodegradable Microspheres Promote Therapeutic Angiogenesis
Source: Adv Healthc Mater. 2024 Sep 11;13(31):2401832. doi: 10.1002/adhm.202401832 (PMC11650400; doi:10.1002/adhm.202401832)
Supplement: Supplementary file 1 — Supporting Information [file ADHM-13-0-s001.docx]

Supporting Information

**Hierarchically Structured Biodegradable Microspheres Promote Therapeutic Angiogenesis**

*Eseelle K. Hendow, Francesco Iacoviello, Mar Ester Casajuana, Caroline Pellet-Many, Richard M. Day**

**Figure S1.** Human interleukin 12 (IL-12 p70) secretion from M1-like and M2-like macrophages after 48 hr incubation in differentiation medium. Increased secretion of IL-12 p70 was used to confirm polarisation towards M1-like macrophages. Data analysed using Mann-Whitney test (*** p<0.001).
